# Matrix Protein 2 Vaccination and Protection against Influenza Viruses, Including Subtype H5N1

**DOI:** 10.3201/eid1303.061125

**Published:** 2007-03

**Authors:** Stephen Mark Tompkins, Zi-Shan Zhao, Chia-Yun Lo, Julia A. Misplon, Teresa Liu, Zhiping Ye, Robert J. Hogan, Zhengqi Wu, Kimberly A. Benton, Terrence M. Tumpey, Suzanne L. Epstein

**Affiliations:** * Food and Drug Administration, Bethesda, Maryland, USA; † University of Georgia, Athens, Georgia, USA; ‡ Centers for Disease Control and Prevention, Atlanta, Georgia, USA; 1 Current affiliation: University of Georgia, Athens, Georgia, USA

**Keywords:** DNA vaccines, M2 protein, influenza A virus H1N1 subtype, influenza A virus H3N2 subtype, influenza A virus H5N1 subtype, research

## Abstract

Changes in influenza viruses require regular reformulation of strain-specific influenza vaccines. Vaccines based on conserved antigens provide broader protection. Influenza matrix protein 2 (M2) is highly conserved across influenza A subtypes. To evaluate its efficacy as a vaccine candidate, we vaccinated mice with M2 peptide of a widely shared consensus sequence. This vaccination induced antibodies that cross-reacted with divergent M2 peptide from an H5N1 subtype. A DNA vaccine expressing full-length consensus-sequence M2 (M2-DNA) induced M2-specific antibody responses and protected against challenge with lethal influenza. Mice primed with M2-DNA and then boosted with recombinant adenovirus expressing M2 (M2-Ad) had enhanced antibody responses that cross-reacted with human and avian M2 sequences and induced T-cell responses. This M2 prime-boost vaccination conferred broad protection against challenge with lethal influenza A, including an H5N1 strain. Vaccination with M2, with key sequences represented, may provide broad protection against influenza A.

## Abstract 2

Vaccination of mice with influenza matrix protein 2 induced cross-reactive antibody responses.

Yearly development of influenza vaccines that are antigenically matched to circulating strains pose extraordinary challenges. A rapidly developing pandemic would shorten the time for strain identification and vaccine preparation; meanwhile, antigenic changes would continue. Moreover, the need to immunize an entirely naive population would exacerbate problems with vaccine production and supply.

Vaccines based on conserved antigens would not require prediction of which strains would circulate during an approaching season and could avoid hurried manufacturing in response to outbreaks. Test vaccination with DNA constructs that express conserved influenza A nucleoprotein (NP) or NP plus matrix (M) induced antibody and T-cell responses and protected against heterosubtypic viruses (*1**,**2*). Despite the virulence and rapid kinetics of challenge infection, DNA vaccination with NP and M achieved limited protection against an H5N1 virus strain isolated from the 1997 human outbreak in Hong Kong (*3*).

The M gene of influenza A encodes 2 proteins, both highly conserved: M1, the capsid protein, and M2, an ion channel protein. M2 contains a small ectodomain (*4*), M2e, which makes it a target for antibody-based immunity. The ability of anti-M2 monoclonal antibody (MAb) to reduce viral replication (*5*) implicates M2, in particular M2e, as a vaccine target. M2 vaccine candidates that have been explored include peptide-carrier conjugates (*6*), baculovirus-expressed M2 (*7*), fusion proteins (*8**,**9*), multiple antigenic peptides (*10*), and M DNA constructs that potentially express M2 (*11**,**12*). In those studies, mice were protected against challenge with homologous or heterosubtypic viruses, but even the heterosubtypic viruses had an M2e sequence identical to the vaccine constructs or differed by only 1 amino acid.

Although most human influenza viruses of H1, H2, or H3 subtypes share identity with the M2e consensus sequence (M2e-con) (*9**,**13*), some influenza A viruses do not. In a study of M2e-carrier conjugate vaccines, serum antibodies specific for M2e-con or M2e-A/PR/8/34 (H1N1) did not cross-react with M2e peptides from H5 and H7 subtype avian viruses that have 3 or 4 mismatches (*6*). In another study, monoclonal and polyclonal antibodies reacted with a subset of avian sequences (*14*). Although a recent study used M2e peptide-liposome vaccines of subtypes including H5N1 with matched challenge viruses (*15*), no prior work has documented protection against challenge with influenza viruses in which M2e sequences differed substantially from those of the immunizing antigen.

Priority is being given to developing vaccines that offer broad protection against multiple influenza subtypes, including H5N1. Indeed, development of conserved-antigen vaccines, and specifically M2-based vaccines, is part of the US Department of Health and Human Services Pandemic Influenza Plan (www.hhs.gov/pandemicflu/plan/). We therefore evaluated M2-based vaccine efficacy against divergent challenge viruses.

## Methods

### Mice

Female BALB/cAnNCR mice were purchased from Division of Cancer Treatment, National Cancer Institute, Frederick, Maryland, USA. The institutions’ Animal Care and Use Committees approved all protocols for animal experiments.

### Viruses

Influenza viruses used were A/PR/8/34 (H1N1) (*3*), A/FM/1/47-MA (H1N1) (*16*), and A/Thailand/SP-83/2004 (H5N1) (*17*). Some virus stocks were propagated in the allantoic cavity of embryonated hen eggs at 34°C for 48–72 h (A/PR/8) or 37°C for 24 h (SP-83). A/FM was prepared as a pooled homogenate of lungs from BALB/c mice infected 4 days previously. All experiments with H5H1 subtypes were conducted under biosafety level 3, enhanced containment.

### Peptides and Peptide Conjugates

M2e 2–24 peptides (no NH2-terminal methionine) were synthesized with COOH-terminal cystine residue and conjugated to maleimide-activated keyhole limpet hemocyanin (KLH) for vaccines. The same peptides were also synthesized without COOH-terminal cystine and used for antibody and T-cell assays. Influenza A NP147–155 and M2e peptides were synthesized in the core facility of the Center for Biologics Evaluation and Research, US Food and Drug Administration. Severe acute respiratory syndrome (SARS) matrix peptide (209–221) was provided by the National Institutes of Health.

### Vectors

Plasmid and recombinant adenoviral (rAd) vectors that express B/NP and A/NP have been described (*18*), as has the plasmid containing the entire M gene of A/PR/8 (*2*). The plasmid VR1012-M2 (termed M2-DNA above) was generated as follows. The plasmid pCR3-M2 was derived by PCR from the vector pCR3-M previously generated from A/PR/8 virus by reverse transcription–PCR (*19*). To modify the sequence to the widely shared M2e sequence, full-length consensus M2 cDNA with Kozak sequence at its 5′ end was generated from 2 overlapping M2 DNA fragments and subcloned into VR-1012, obtained under material transfer agreement from Vical, Inc., San Diego, CA, USA. The sequence of the M2 insert was confirmed by restriction digestion and sequence analysis. The replication-incompetent adenovirus that expressed the M2 protein with the consensus sequence (M2-Ad) was constructed by using Gateway cloning and the ViraPower Adenoviral Expression System (both by Invitrogen, Carlsbad, CA, USA) according to manufacturer’s instructions. Briefly, the M2 cDNA from VR1012-M2 was cloned by PCR into the pENTR/D-TOPO Gateway vector and then transferred into the pAd/CMV/V5-DEST adenoviral Gateway vector by LR Clonase (Invitrogen) reaction to give pAd/CMV-M2. Integrity and proper insertion of the cloned M2 cDNA were confirmed by sequencing. M2-Ad was generated by transfection of 293A cells with pAd/CMV-M2. M2 expression was confirmed by immunohistochemical staining of M2-Ad-infected Madin-Darby canine kidney cells with M2-specific polyclonal sera (data not shown). High-titered stocks of rAd were prepared by ViraQuest, Inc. (North Liberty, IA, USA). Adenovirus stocks were stored in 3% sucrose/phosphate-buffered saline (PBS) at 1–2×10^12^ particles/mL and confirmed as negative for replication-competent adenovirus by passage on nonpermissive cells.

### Immunization

Mice were given an intraperitoneal injection of 40 μg peptide-KLH or unconjugated KLH in complete Freund’s adjuvant (emulsified 1:1 with antigen in PBS). Three weeks later, the mice were given an intraperitoneal booster injection with peptide-KLH in incomplete Freund’s adjuvant; 13 days later blood was collected. Injections were started at 8–10 weeks of age for peptide and 6 weeks of age for DNA. DNA vaccination at doses of 50 μg/mouse (unless noted otherwise in a figure legend) in low-endotoxin PBS (AccuGENE, Cambrex, East Rutherford, NJ, USA) was given intramuscularly in the quadriceps, half to each leg, in 3 doses 2 weeks apart. In some experiments, mice were given a booster injection of rAd intramuscularly at a dose of 10^10^ particles/mouse, 2–3 weeks after the last dose of DNA.

### Challenge

Challenge virus in 50 μL of PBS was administered intranasally to anesthetized mice. Isoflurane or ketamine/xylazine was used for mice challenged with H1N1 subtype. Reported 50% lethal dose (LD_50_) for H1N1 subtype was determined for 8-week-old naive BALB/c mice for each anesthetic (and may vary from the actual LD_50_ for the older vaccinated mice that were challenged). Subtype H5N1 was administered intranasally to mice anesthetized with 2,2,2-tribromoethanol in tert-amyl alcohol (Avertin; Aldrich Chemical Co., Milwaukee, WI, USA). Some mice were humanely killed so their lungs could be harvested; others were monitored for body weight and death. Monitoring continued until all animals died or were recovering, as indicated by body weight.

### In Vivo T-Cell Depletion

Acute depletion of lymphocyte populations by MAb treatment on days –3, +2, +8 relative to day of challenge was performed as described previously (18) and used MAbs GK1.5, specific for mouse CD4; 2.43, specific for mouse CD8; and SFR3-DR5, specific for a human leukocyte antigen as a negative control. Splenocytes were analyzed 2 days after challenge (before the next injection) by flow cytometry to confirm completeness of in vivo T-cell depletion, as described (*2*).

### ELISA

ELISA for M2-specific antibodies was performed on plates coated with 15 µg/mL of synthetic peptides in 0.007 mol/L borate buffer and 0.025 mol/L saline; the rest of the procedure was as described (*20*).

### Passive Serum Transfer

Naive mice were given intraperitoneal injections of pooled serum, 1 mL per mouse, from mice immunized with M2-DNA plus matched Ad booster or from control mice (B/NP-DNA, M2-H5(HK) peptide-KLH conjugate, or A/PR/8 virus). Mice were challenged with a moderate dose of A/PR/8 virus the day after serum transfer; antibody levels in recipients were not measured.

### Spleen Cell Fractionation

T cells were enriched by negative selection that used magnetic beads. Briefly, splenocytes were depleted of erythrocytes and labeled with biotinylated antimouse B220, CD11b, and PanNK antibodies (BD Pharmingen, San Diego, CA, USA). After labeling, cells were incubated with Streptavidin MicroBeads (Miltenyi Biotec, Auburn, CA, USA). Unlabeled T cells and labeled non–T cells were separated through the Miltenyi AutoMACS system according to the manufacturer’s instructions.

### Enzyme-linked Immunosorbent Spot (ELISPOT) Assay

This assay detected T-cell responses to M2 peptides. ELISPOT IP plates (Millipore; Billerica, MA, USA) were coated with 50 µL of Hank’s balanced salt solution (Hyclone, Logan, UT, USA) containing 5 µg/mL of anti–interferon-γ (IFN-γ) MAb AN18 (BD Pharmingen) and incubated overnight at 4°C. The membrane was washed and then blocked with medium containing 10% fetal bovine serum for 60–90 min at room temperature. Splenocytes depleted of erythrocytes were added to wells in 2-fold dilutions, starting at 250,000 cells/well in 50 µL. Peptides (SARS-M-209–221, NP147–155 of A/PR/8, or M2–2-24 of A/PR/8) were added at a final concentration of 1 µg/mL. After incubation for 36–48 h at 37°C, bound IFN-γ was detected with 50 µL of biotinylated MAb R4–6A2 (BD Pharmingen) at 1 µg/mL. Spots were developed by using alkaline phosphatase–labeled streptavidin and 5-bromo, 4-chloro, 3-indolylphosphate/nitroblue tetrazolium substrate (Kirkegaard and Perry Laboratories, Gaithersburg, MD, USA) and counted with an ELISPOT reader (Zeiss; Thornwood, NY, USA).

### Virus Quantitation

Lungs were homogenized in 1 mL of sterile PBS, clarified by centrifugation, and titrated for virus infectivity by 50% egg infectious dose (EID_50_) assay as described (*3*). The limit of virus detection is 1.2 log_10_ EID_50_/mL. Challenge virus stocks were titrated on Madin-Darby canine kidney cells as described previously (*18*).

### Statistical Analysis

The serologic assays and detection of M2-specific T cells were performed multiple times with comparable results. Some vaccinations were repeated with independent groups (as noted in the figure legends); those performed once used numbers of animals per group adequate for statistical significance. Lung virus titers were compared by using 1-way analysis of variance on log-transformed data, followed by pairwise multiple comparison (Holm-Sidak method). Weight loss after challenge was compared for survivors on each day by also using 1-way analysis of variance followed by pairwise multiple comparison (Holm-Sidak). This method overestimates body weight in groups with deaths because the animals that died would have had very low body weights, affecting the average, especially in the negative control groups. Nonetheless, differences between vaccinated groups were significant in the instances stated. Comparison of cumulative survival rates used the log-rank test, followed by pairwise multiple comparison, again by using the Holm-Sidak method. Overall significance level for Holm-Sidak tests was p = 0.05. All statistical analyses were performed with SigmaStat Software v3.11 (Systat Software, Point Richmond, CA, USA).

## Results

### M2e-KLH Vaccination

**Figure 1:**
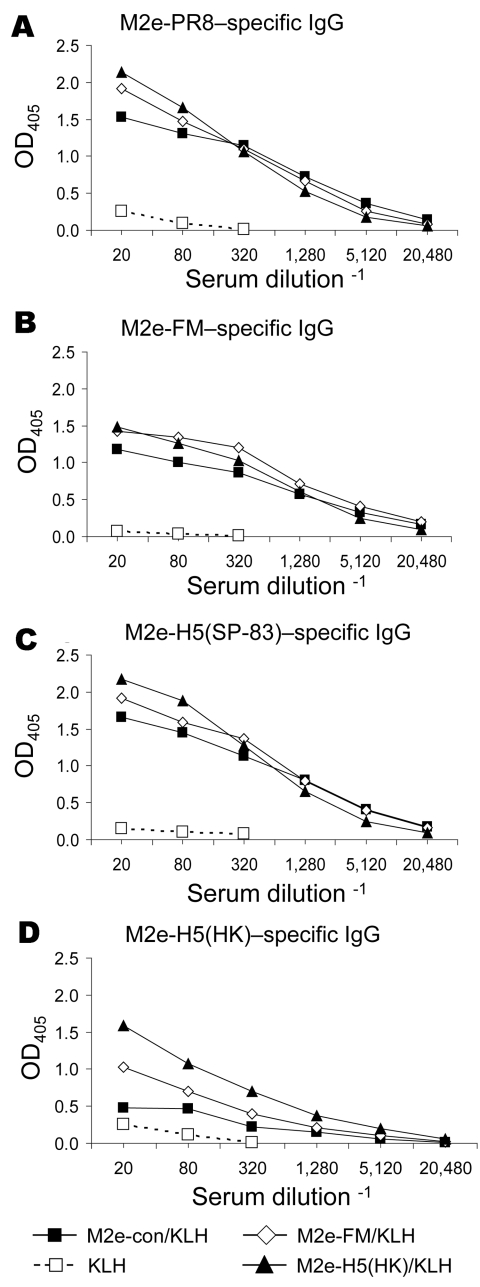
Results of matrix protein 2 (M2)e–keyhole limpet hemocyanin (KLH) vaccination, showing induction of cross-reactive antibody responses. Mice (7–9 per group) were immunized intraperitoneally with KLH or M2e peptides conjugated to KLH (M2e-con/KLH, M2e-FM/KLH, or M2e-H5(HK)/KLH) in complete Freund’s adjuvant. After 21 days, the mice were given an intraperitoneal booster with KLH or M2e-peptide/KLH in incomplete Freund’s adjuvant. Immune serum was collected 13 days after booster and assayed for immunoglobulin (Ig) G reactive to various M2e peptides by ELISA. Plates were coated with M2e-PR8 (panel A), M2e-FM (panel B), M2e-H5(SP-83) (panel C), or M2e-H5(HK) (panel D). Data are representative of multiple experiments. OD, optical density; e, ectodomain.

For proof-of-concept studies, peptides representing M2e-con (*9*) and additional viral M2 ectodomains (Table) were conjugated to KLH and used to immunize BALB/c mice. Immune serum samples were analyzed for M2-specific antibodies by ELISA on plates coated with synthetic M2e peptides with sequences from 2 viruses of H1N1 and 2 of H5N1 subtype. Serum from KLH-immune mice did not react with any of the peptides, but each M2e-immune serum sample reacted with M2e-PR8, M2e-FM, and M2e-H5(SP-83) peptides (Figure 1 A–C). Cross-reactions were lower on the M2e-H5(HK) peptide (Figure 1D).

**Table:** Sequences of matrix protein 2 (M2) ectodomains

Strain	Abbreviation	Subtype	M2e Sequence*†
Consensus	M2e-con	‡	MSLLTEVETPIRNEWGCRCNDSSD
A/PR/8	M2e-PR8	H1N1	MSLLTEVETPIRNEWGCRCN**G**SSD
A/FM/1/47-MA	M2e-FM	H1N1	MSLLTEVETP**TK**NEW**E**CRCNDSSD
A/HK/156/97	M2e-H5(HK)	H5N1	MSLLTEVET**LT**RN**G**WGCRC**S**DSSD
A/Thailand/SP-83/04	M2e-H5(SP-83)	H5N1	MSLLTEVETP**T**RNEW**E**CRC**S**DSSD

*Variations from the M2e consensus sequence are in **boldface.** †Virus sequences available at www.ncbi.nlm.nih.gov/genomes/FLU and www.flu.lanl.gov ‡Consensus sequence derived from human influenza viruses of H1, H2, and H3 subtypes (*9**,**13*).

**Figure 2:**
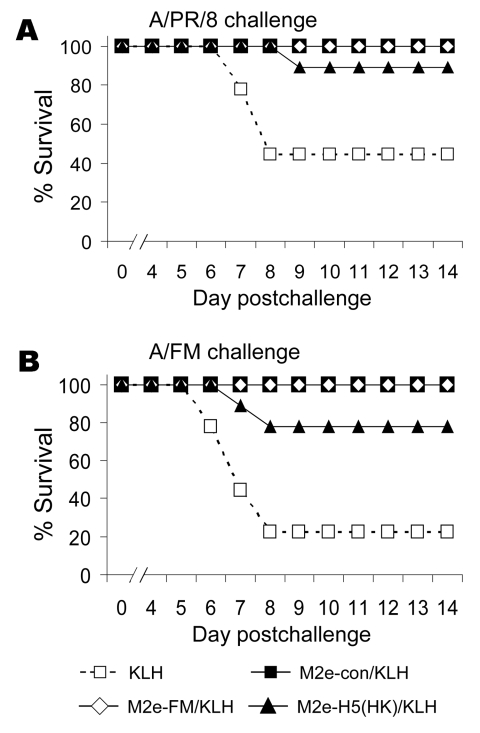
Results of matrix protein 2 (M2)e–keyhole limpet hemocyanin (KLH) vaccination, showing cross-protection. Mice (7–9 per group) were vaccinated as in Figure 1. Six weeks after the booster, they were anesthetized with isoflurane and challenged with 10x the 50% lethal dose (LD_50_). Of A/PR/8 (A) or A/FM (B) viruses and then monitored for survival. Cumulative survival rates after challenge with A/PR/8 or A/FM virus differed significantly from those of KLH controls for all M2e-conjugates (p = 0.001 and p<0.001, respectively, log-rank). e, ectodomain.

M2e-vaccinated mice were then challenged with either A/PR/8 or A/FM and monitored for weight loss (as a measure of illness) and death. Weight losses were consistent with a hierarchy of protection based on sequence similarity (data not shown), and differences from control mice were statistically significant. In the same groups, 100% of mice vaccinated with M2e-con or M2e-FM survived challenge with A/PR/8 and A/FM, but M2e-H5(HK)/KLH vaccination provided incomplete protection (Figure 2A,B).

### M2-DNA Vaccination

**Figure 3:**
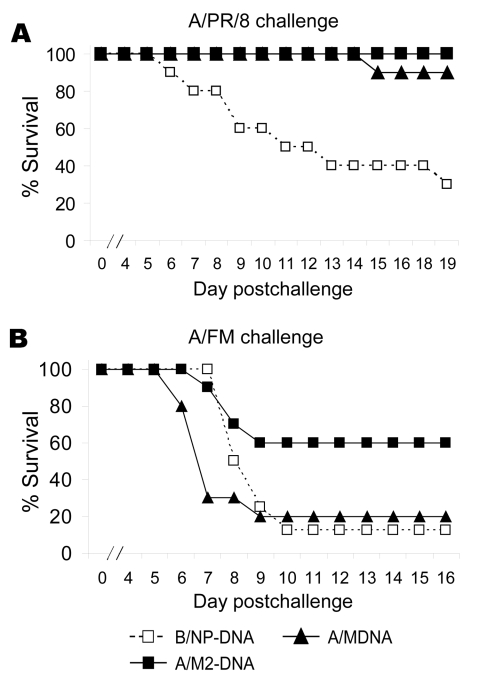
Results of matrix protein 2 (M2)–DNA vaccination, showing protection against divergent influenza viruses. Mice (8–10 per group) were vaccinated with DNA as described in Methods except at a dose of 100 μg/mouse. Approximately 2 weeks after the last dose of DNA, mice were challenged with 7× the 50% lethal dose (LD_50_) of virus and monitored for survival. A) A/PR/8 challenge: Cumulative survival rate of mice vaccinated with M-DNA or M2-DNA was significantly higher than that of mice vaccinated with B/NP-DNA (p<0.001, log rank). B) A/FM challenge: Cumulative survival rate differed significantly among groups (p = 0.041, log-rank), although in post hoc Holm-Sidak tests, pairs did not differ significantly (p≥0.05).

To investigate whether, like M2e peptide, DNA vaccination could protect against viruses with quite divergent M2e sequence, we tested M- and M2-DNA for efficacy. M-DNA with the A/PR/8 sequence protected against challenge with A/PR/8 (Figure 3A). M2-DNA with the consensus sequence also protected against this A/PR8 challenge; however, M1-DNA did not (data not shown). For challenge with A/FM, M2-DNA was more efficacious than M-DNA (Figure 3B).

### M2-DNA Vaccination Followed by M2-Ad Boost

**Figure 4:**
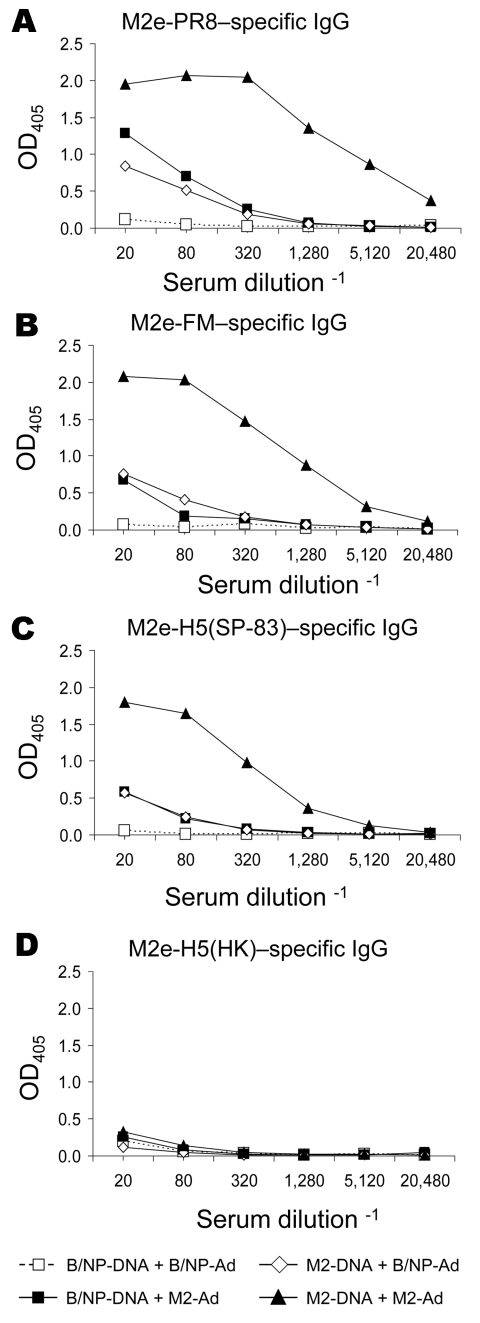
Results of matrix protein 2 (M2) vaccination and booster with DNA prime–adenovirus (Ad), showing cross-reactive antibodies. Mice (8–10 per group) were vaccinated with DNA and given an Ad booster as described in Methods. Immune serum collected 3 weeks after the booster was assayed for immunoglobulin (Ig) G reactive to various M2e peptides by ELISA, as described in Methods. Plates were coated with M2e-PR8 (panel A), M2e-FM (panel B), M2e-H5(SP-83) (panel C), or M2e-H5(HK) (panel D). OD, optical density.

Previously, we have shown for NP vaccination that boosting with rAd induces more potent antibody and T-cell (especially CD8^+^) responses than does DNA vaccination alone and can protect against challenge with highly pathogenic H5N1 subtype (18). We investigated whether boosting with rAd would also enhance immunity to M2. The A/M2 gene with the consensus sequence was cloned into a replication-deficient Ad construct (M2-Ad). Mice were primed with DNA as before and boosted with M2-Ad or control B/NP-Ad. Two weeks later, serum samples were collected and assayed for M2-specific immunoglobulin (Ig) G by ELISA on peptides as above (Figure 4A–D). Mice given the booster of M2-DNA+M2-Ad had dramatically greater M2e-PR8–specific IgG antibody responses than mice given either component alone (M2-DNA+B/NP-Ad or B/NP-DNA+M2-Ad; Figure 4A). Moreover, IgG cross-reactivity with M2e-FM (3 amino acid differences; Figure 4B) and M2e-H5(SP-83) (3 amino acid differences; Figure 4C) was found with serum from mice given M2-Ad; cross-reactivity was even greater with serum from mice given M2-DNA+M2-Ad. These serum samples, however, did not cross-react with M2e-H5(HK) (4 amino acid differences; Figure 4D), although serum from mice immunized with M2e-H5(HK)-KLH as positive controls reacted strongly (data not shown).

**Figure 5:**
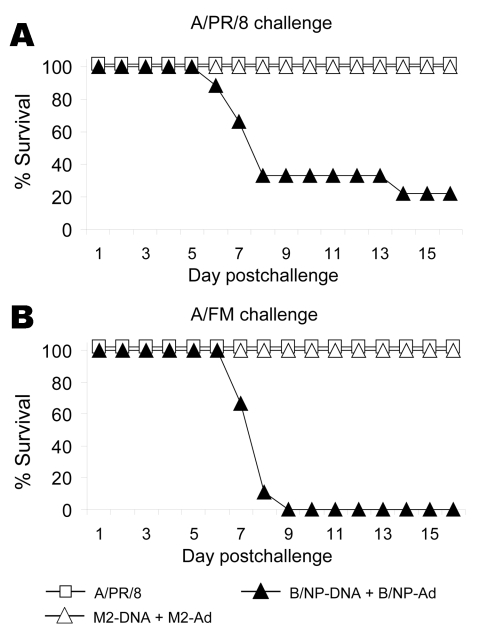
Results of vaccination and booster with DNA prime–adenovirus (Ad), showing cross-protection. Mice (8–10 per group) were immunized as in Figure 4 or intranasally given a sublethal priming infection with A/PR/8. Three weeks later they were challenged with a high dose of A/PR/8 (1.5x 10^4^ 50% lethal dose [LD_50_]) or moderate dose of A/FM (10 LD_50_) and monitored for survival. The cumulative survival rate for mice immunized with A/PR/8 and M2-DNA+M2-Ad was significantly higher than that for mice immunized with B/NP-DNA+B/NP-Ad (p<0.001, log rank). Data are representative of multiple experiments.

Protective immunity due to vaccination with M2-DNA+M2-Ad was tested by challenge with A/PR/8 (high-dose) or A/FM (moderate dose) virus. Of mice vaccinated with M2-DNA+M2-Ad, 100% survived challenge with A/PR/8 and A/FM virus; of mice vaccinated with B/NP-DNA+B/NP-Ad, 20% survived challenge with A/PR/8 and none survived challenge with A/FM (Figure 5A,B). Thus, the prime-boost vaccination protected against challenge viruses in which M2e sequences were similar to or divergent from those of the vaccine.

### T-cell Response

**Figure 6:**
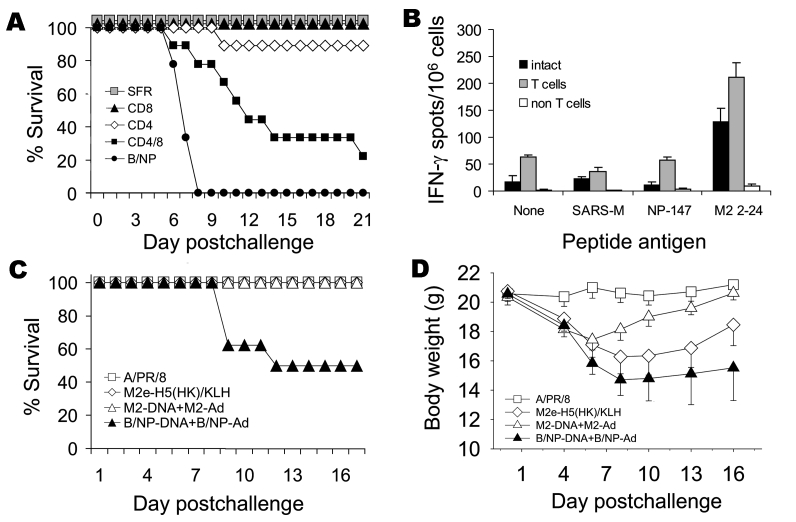
Role of T- and B-cell immunity in matrix protein 2 (M2)–specific protective immunity. A) Mice (9 per group) were immunized with M2-DNA or B/NP-DNA and boosted with matched adenovirus (Ad) as described in Methods. Three weeks after Ad boost, M2-DNA groups were acutely depleted of T cells with monoclonal antibodies (MAbs) to CD4^+^ or CD8^+^ or both, or given control MAb SFR3-DR5, as described in Methods. Mice were challenged with 1.5× 10^4^ 50% lethal doses (LD_50_) of A/PR/8. Compared with the cumulative survival rate for the SFR control, survival rates differed significantly for mice depleted of both T-cell subsets (p<0.001, log-rank), although some protection remained, which differed significantly from that of the B/NP control (p<0.001, log-rank). B) Mice were immunized with M2-DNA+M2-Ad as described under A. Five months after mice received the Ad boost, spleen cells were isolated and pooled from immune mice (n = 10), fractionated into T-cell and non–T-cell populations, and assayed for interferon-γ (IFN-γ)–producing cells by enzyme-linked immunosorbent spot assay, as described in Methods. C and D) Serum collected from immune mice was passively transferred intraperitoneally into naive BALB/c mice (8 per group). The recipients were challenged with 10 LD_50_ of A/PR/8 and monitored for survival (C) and weight loss (D). The cumulative survival rate for mice given A/PR/8 immune serum, M2-DNA+M2-Ad-immune serum, or M2e-H5(HK)/keyhole limpet hemocyanin–immune serum was significantly higher than that for mice given B/NP-DNA+B/NP-Ad–immune serum (p<0.001, log rank). For weight loss, M2 prime-boost differed from B/NP prime-boost at days 8, 10, and 13 (p≤0.003, analysis of variance; p<0.05, Holm-Sidak pairwise multiple comparison).

T-cell responses to M2 have been observed (*7*). Immunization with cDNA expressing full-length M2 protein might induce, in addition to antibody, M2-specific T-cell responses not induced by peptides. To address the contribution of T-cell responses, we immunized mice to M2 by prime-boost and acutely depleted them of T cells just before and during the challenge period. Lymphocyte depletion was confirmed to be complete; residual CD4^+^ or CD8^+^ cells were <1% (data not shown). Of the B/NP control mice, 100% died of A/PR/8 infection by day 8 after challenge, while 100% of M2-immune mice treated with a control MAb (SFR) survived (Figure 6A). Individual depletion of CD4^+^ or CD8^+^ T cells did not abrogate protection. Depletion of CD4^+^ and CD8^+^ T cells together partially, but statistically significantly, abrogated M2-induced protection (Figure 6A) but left some protection significantly different from that in the B/NP control mice. Thus, under these challenge conditions, T cells are important. M2e-specific immunity has been reported to be natural killer (NK)-cell dependent (*21*). We found that mice depleted of NK cells with anti–asialo-GM1 antibody were protected similarly to controls (data not shown). Thus, while NK cells may play a role, they were not required under the conditions we studied.

Given the effect of T-cell depletion, we tested for in vitro M2-specific IFN-γ–producing T cells. The IFN-γ ELISPOT assay showed positive responses to an amino-terminal M2 peptide in spleen cells from mice immune to M2-DNA+M2-Ad (Figure 6B) and to A/PR/8 (data not shown). After spleen cells were fractionated by magnetic bead separation (see Methods), the non–T-cell fraction did not respond, while the unfractionated cells and T-cell fraction maintained a strong M2-specifc IFN-γ response (Figure 6B).

### Role of M2-specific Antibody

On the basis of information from previous studies (see Discussion), we tested the ability of antibodies induced by DNA prime–Ad boost to passively transfer protection. All (100%) mice given serum from M2-immune or A/PR/8-infected mice survived, while only 50% given control B/NP-immune serum survived (Figure 6D; p<0.001, log rank). Passive serum antibody from M2 prime–boost immune mice also conferred significant protection against weight loss (Figure 6D).

### Heterologous Challenge, Including SP-83 (H5N1)

**Figure 7:**
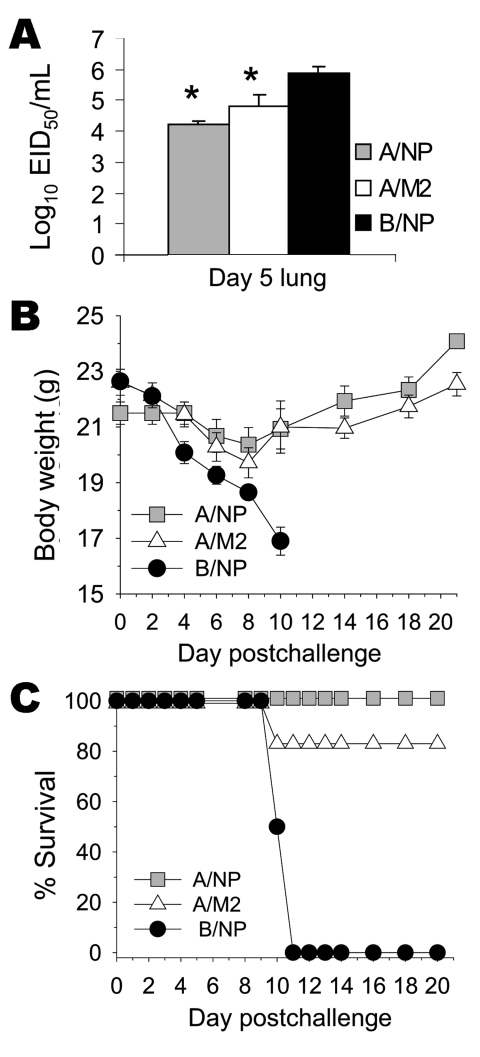
Results of vaccination with matrix protein 2 (M2)–DNA plus M2-adenovirus (Ad) and challenge with heterologous H5N1 subtype. Mice (10 per group) were vaccinated with A/NP-DNA, M2-DNA, or B/NP-DNA and boosted with matched Ad, as described in the Methods. Seventeen days after Ad boost, mice were challenged with 10× 50% lethal dose (LD_50_) of SP-83 (H5N1). A random subset pf mice (4/group) were humanely killed on day 5, and their lungs were assayed for virus titer, as described in the Methods. A). Remaining mice were monitored for weight loss (B) and survival (C). The cumulative survival rates for A/NP and M2 immune mice were significantly higher than those for B/NP-immune mice but did not differ from each other significantly (p<0.001, log-rank, Holm-Sidak pairwise comparison: p<0.05 comparing B/NP with A/NP or M2 groups, p≥0.05 comparing A/NP and M2 groups). *Lung virus titers in A/NP- and M2-immune mice were significantly lower than in B/NP-immune mice but did not differ from each other significantly (p = 0.004, analysis of variance. Holm-Sidak pairwise comparison: p<0.05 comparing B/NP with A/NP or M2 groups, p≥0.05 comparing A/NP and M2 groups). SARS, severe acute respiratory syndrome; IFN-γ, interferon-γ.

Because protection against challenge with A/FM virus that has an M2e sequence quite divergent from that of the immunizing sequence was encouraging, we tested whether M2-DNA+M2-Ad vaccination could protect against challenge with H5N1 subtype. Mice were immunized 3× with B/NP-DNA (negative control), A/NP-DNA (positive control), or consensus M2-DNA, and boosted with matched rAd. Mice were challenged with a lethal dose of A/Thailand/SP-83/2004 (H5N1) virus in which M2e differed from the consensus by 3 amino acids. On day 5 after infection, a random subset of animals was killed and their lung virus titers were measured. Virus titers of mice vaccinated with A/NP or with M2 were significantly reduced compared with those of control mice (Figure 7A). The remaining mice were monitored for weight loss and survival. Weight loss was less in mice vaccinated with A/NP and M2 than in control mice (Figure 7B). All the B/NP-immune mice died of SP-83 infection by day 11 postchallenge. All (100%) A/NP-immune mice and all but 1 M2-immune mouse survived (Figure 7C).

## Discussion

Our results indicate that M2 vaccination can induce cross-reactive antibody responses, virus-specific T-cell responses, and protection against challenge with lethal heterologous virus. As has been found in previous studies of M2-based vaccines, we found strong antibody responses to the conserved M2e region. Anti-M2 antibodies in the serum of mice immunized with M DNA suggest expression of M2 from this plasmid (not shown). However, reactivity could be due to the 9 amino acid portion shared with the M1 sequence, and we did not explore this possibility. Fan et al. used A/PR/8 and Aichi M2e-carrier conjugates for immunization, and the resulting antibodies did not cross-react with the avian M2e sequences tested (*6*). We found cross-reactivity with M2e peptides that had considerable sequence divergence from the human influenza M2 consensus. Immunization with M2e-con/KLH induced antibodies that were reactive with M2e-H5(SP-83) peptide but less reactive with M2e-H5(HK). However, immunization with M2e-H5(HK)/KLH induced antibodies that were reactive with all the M2e peptides. This pattern parallels the results of Liu et al. (*14*). However, neither Fan et al. nor Liu et al. investigated protection against challenge with H5N1 subtype.

In our lethal challenge studies, M2e peptide conjugates protected against not only a 1934 subtype H1N1 virus (A/PR/8) but also a 1947 subtype H1N1 virus (A/FM). The latter virus, which is virulent in mice, has an M2e sequence with 3 amino acid differences from the consensus and thus is as divergent from the consensus sequence as some M2-H5 sequences. Encouraged by this broad cross-reactivity and cross-protection, we expanded the study to DNA vaccination and DNA prime–Ad boost regimens. These approaches have the advantage of providing more epitopes than peptide immunization and relevant T-cell immunity.

Using M2 consensus DNA vaccination with or without Ad boost, we again saw cross-reactivity on avian peptides M2e-H5(SP-83) and M2e-H5(HK), although cross-reactivity was low on the HK peptide. T-cell responses to M2 peptides were detected by ELISPOT.

Several studies have shown that M2e-specific antibodies can mediate protection against influenza infection in vivo (*9*,*10*,*13*). In agreement with those studies, we found that serum antibodies induced by peptide conjugates or by prime-boost vaccination could transfer protection to naive recipients. We found that T cells were also important because depletion of CD4^+^ and CD8^+^ T cells during the challenge period reduced protection against a higher challenge dose. This could reflect M2e-specific memory T cells, which we have demonstrated in spleen and peripheral blood by ELISPOT, or a concurrent T-cell response to challenge virus supplementing the protective effects of antibodies.

In lethal challenge studies, the M2 consensus DNA and rAd constructs could protect against not only A/PR/8 but also against A/FM, a virus quite divergent in the M2e sequence. Furthermore, they could protect mice against challenge with SP-83 (H5N1) isolated from a fatal human case, at a dose lethal to control mice. Virus replication in lungs and illness reflected by loss of body weight were also reduced by M2 immunization. Protection against challenge with other H5N1 subtypes remains to be explored, and serologic results on M2e-H5(HK) peptide suggest results of such studies might differ on the basis of sequence variations.

M2 expression constructs with various M2e sequences could be used as vaccines. Our observation of protection across substantial sequence divergence means that H5-derived vaccines might also protect against circulating H1N1 and H3N2 subtypes. An additional advantage of protection across substantial divergence is potential protection by an M2 vaccine against an unexpected subtype that could cause a pandemic.

One concern about M2 vaccines is the possibility of escape mutants. A study of forced escape mutants found limited diversity (*13*), which indicates that structural constraints, perhaps due to requirements of the M1 structure encoded by the same segment, may limit drift.

The cross-reactivity and protective efficacy of M2-specific antibodies suggest that M2-specific MAbs could be useful for antiviral therapy. These features, combined with constraints on M2 structure, highlight the potential of M2-specific MAbs to inhibit replication of influenza viruses, including some H5N1 strains. Although traditional M2-directed drugs (e.g., amantadine) have led to drug resistance, the mutations that confer resistance are within the transmembrane region (*22*), which may have fewer structural constraints than the ectodomain.

An M2 prime-boost regimen is intended to be combined with vaccination against additional antigens rather than acting as a standalone vaccine. For example, prime-boost vaccination against conserved NP is highly protective ([*18*]; Figure 7). The use of multiple antigens has several advantages: reduced likelihood of escape mutants, better coverage of human leukocyte antigen haplotypes in the genetically diverse human population, and a broader spectrum of immune response mechanisms (with antibodies perhaps dominating for M2 and cytotoxic T lymphocytes for NP).

Vaccines based on conserved antigens are not intended to replace strain-matched vaccines that induce neutralizing antibodies and thus prevent infection. However, strain-matched vaccines may be difficult to produce in adequate quantities in short time periods, and continued antigenic drift may render them ineffective. Vaccinations as described here, based on M2, might reduce deaths and severity of disease while strain-matched vaccines were being prepared and could enhance protection afforded by inactivated vaccines. Immunogenicity and safety studies in people are needed to evaluate this approach.

## References

[1] Ulmer JB, Donnelly JJ, Parker SE, Rhodes GH, Felgner PL, Dwarki VJ, Heterologous protection against influenza by injection of DNA encoding a viral protein. Science. 1993;259:1745–9. 10.1126/science.8456302 8456302

[2] Epstein SL, Stack A, Misplon JA, Lo CY, Mostowski H, Bennink J, Vaccination with DNA encoding internal proteins of influenza virus does not require CD8(+) cytotoxic T lymphocytes: either CD4(+) or CD8(+) T cells can promote survival and recovery after challenge. Int Immunol. 2000;12:91–101. 10.1093/intimm/12.1.91 10607754

[3] Epstein SL, Tumpey TM, Misplon JA, Lo CY, Cooper LA, Subbarao K, DNA vaccine expressing conserved influenza virus proteins protective against H5N1 challenge infection in mice. Emerg Infect Dis. 2002;8:796–801. 12141964 10.3201/eid0808.010476 PMC2732511

[4] Lamb RA, Krug RM. Orthomyxoviridae: the viruses and their replication. In: Knipe DM, Howley, PM, Griffin DE, Martin MA, Lamb RA, Roizman B, et al., editors. Fields Virology. 4th ed. Philadelphia: Lippincott Williams & Wilkins; 2001. p. 1487–503.

[5] Zebedee SL, Lamb RA. Influenza A virus M2 protein: monoclonal antibody restriction of virus growth and detection of M2 in virions. J Virol. 1988;62:2762–72. 2455818 10.1128/jvi.62.8.2762-2772.1988 PMC253710

[6] Fan J, Liang X, Horton MS, Perry HC, Citron MP, Heidecker GJ, Preclinical study of influenza virus A M2 peptide conjugate vaccines in mice, ferrets, and rhesus monkeys. Vaccine. 2004;22:2993–3003. 10.1016/j.vaccine.2004.02.021 15297047

[7] Slepushkin VA, Katz JM, Black RA, Gamble WC, Rota PA, Cox NJ. Protection of mice against influenza A virus challenge by vaccination with baculovirus-expressed M2 protein. Vaccine. 1995;13:1399–402. 10.1016/0264-410X(95)92777-Y 8578816

[8] Frace AM, Klimov AI, Rowe T, Black RA, Katz JM. Modified M2 proteins produce heterotypic immunity against influenza A virus. Vaccine. 1999;17:2237–44. 10.1016/S0264-410X(99)00005-5 10403591

[9] Neirynck S, Deroo T, Saelens X, Vanlandschoot P, Jou WM, Fiers W. A universal influenza A vaccine based on the extracellular domain of the M2 protein. Nat Med. 1999;5:1157–63. 10.1038/13484 10502819

[10] Mozdzanowska K, Feng J, Eid M, Kragol G, Cudic M, Otvos JL, Induction of influenza type A virus-specific resistance by immunization of mice with a synthetic multiple antigenic peptide vaccine that contains ectodomains of matrix protein 2. Vaccine. 2003;21:2616–26. 10.1016/S0264-410X(03)00040-9 12744898

[11] Okuda K, Ihata A, Watabe S, Okada E, Yamakawa T, Hamajima K, Protective immunity against influenza A virus induced by immunization with DNA plasmid containing influenza M gene. Vaccine. 2001;19:3681–91. 10.1016/S0264-410X(01)00078-0 11395202

[12] Watabe S, Xin K-Q, Ihata A, Liu L-J, Honsho A, Aoki I, Protection against influenza virus challenge by topical application of influenza DNA vaccine. Vaccine. 2001;19:4434–44. 10.1016/S0264-410X(01)00194-3 11483269

[13] Zharikova D, Mozdzanowska K, Feng J, Zhang M, Gerhard W. Influenza type A virus escape mutants emerge in vivo in the presence of antibodies to the ectodomain of matrix protein 2. J Virol. 2005;79:6644–54. 10.1128/JVI.79.11.6644-6654.2005 15890902 PMC1112148

[14] Liu W, Zou P, Ding J, Lu Y, Chen YH. Sequence comparison between the extracellular domain of M2 protein human and avian influenza A virus provides new information for bivalent influenza vaccine design. Microbes Infect. 2005;7:171–7. 10.1016/j.micinf.2004.10.006 15777646

[15] Ernst WA, Kim HJ, Tumpey TM, Jansen AD, Tai W, Cramer DV, Protection against H1, H5, H6 and H9 influenza A infection with liposomal matrix 2 epitope vaccines. Vaccine. 2006;24:5158–68. 10.1016/j.vaccine.2006.04.008 16713037

[16] Smeenk CA, Brown EG. The influenza virus variant A/FM/1/47-MA possesses single amino acid replacements in the hemagglutinin, controlling virulence, and in the matrix protein, controlling virulence as well as growth. J Virol. 1994;68:530–4. 8254767 10.1128/jvi.68.1.530-534.1994 PMC236317

[17] World Health Organization Global Influenza Program Surveillance Network. Evolution of H5N1 avian influenza viruses in Asia. Emerg Infect Dis. 2005;11:1515–21. 16318689 10.3201/eid1110.050644 PMC3366754

[18] Epstein SL, Kong WP, Misplon JA, Lo CY, Tumpey TM, Xu L, Protection against multiple influenza A subtypes by vaccination with highly conserved nucleoprotein. Vaccine. 2005;23:5404–10. 10.1016/j.vaccine.2005.04.047 16011865

[19] Huang X, Liu T, Muller J, Levandowski RA, Ye Z. Effect of influenza virus matrix protein and viral RNA on ribonucleoprotein formation and nuclear export. Virology. 2001;287:405–16. 10.1006/viro.2001.1067 11531417

[20] Benton KA, Misplon JA, Lo CY, Brutkiewicz RR, Prasad SA, Epstein SL. Heterosubtypic immunity to influenza A virus in mice lacking IgA, all Ig, NKT cells, or gamma delta T cells. J Immunol. 2001;166:7437–45. 11390496 10.4049/jimmunol.166.12.7437

[21] Jegerlehner A, Schmitz N, Storni T, Bachmann MF. Influenza A vaccine based on the extracellular domain of M2: weak protection mediated via antibody-dependent NK cell activity. J Immunol. 2004;172:5598–605. 15100303 10.4049/jimmunol.172.9.5598

[22] Shiraishi K, Mitamura K, Sakai-Tagawa Y, Goto H, Sugaya N, Kawaoka Y. High frequency of resistant viruses harboring different mutations in amantadine-treated children with influenza. J Infect Dis. 2003;188:57–61. 10.1086/375799 12825171

